# Analyzing implementation dynamics using theory-driven evaluation principles: lessons learnt from a South African centralized chronic dispensing model

**DOI:** 10.1186/s12913-017-2640-2

**Published:** 2017-12-04

**Authors:** Bvudzai Priscilla Magadzire, Bruno Marchal, Tania Mathys, Richard O. Laing, Kim Ward

**Affiliations:** 1 School of Public Health, University of the Western Cape, Bellville, South Africa; 2 Department of Public Health, Institute of Tropical Medicine, Antwerp, Belgium; 3 Western Cape Department of Health, Cape Town, South Africa; 4 School of Public Health, Department of Global Health, Boston University, Boston, MA USA; 5 School of Pharmacy, University of the Western Cape, Bellville, South Africa

**Keywords:** Chronic Dispensing Unit, Centralized dispensing, Medicines supply chain, theory-driven evaluation, Access to medicines, Western Cape, South Africa

## Abstract

**Background:**

Centralized dispensing of essential medicines is one of South Africa’s strategies to address the shortage of pharmacists, reduce patients’ waiting times and reduce over-crowding at public sector healthcare facilities. This article reports findings of an evaluation of the Chronic Dispensing Unit (CDU) in one province. The objectives of this process evaluation were to: (1) compare what was planned versus the actual implementation and (2) establish the causal elements and contextual factors influencing implementation.

**Methods:**

This qualitative study employed key informant interviews with the intervention’s implementers (clinicians, managers and the service provider) [*N* = 40], and a review of policy and program documents. Data were thematically analyzed by identifying the main influences shaping the implementation process. Theory-driven evaluation principles were applied as a theoretical framework to explain implementation dynamics.

**Results:**

The overall participants’ response about the CDU was positive and the majority of informants concurred that the establishment of the CDU to dispense large volumes of medicines is a beneficial strategy to address healthcare barriers because mechanical functions are automated and distribution of medicines much quicker. However, implementation was influenced by the context and discrepancies between planned activities and actual implementation were noted. Procurement inefficiencies at central level caused medicine stock-outs and affected CDU activities. At the frontline, actors were aware of the CDU’s implementation guidelines regarding patient selection, prescription validity and management of non-collected medicines but these were adapted to accommodate practical realities and to meet performance targets attached to the intervention. Implementation success was a result of a combination of ‘hardware’ (e.g. training, policies, implementation support and appropriate infrastructure) and ‘software’ (e.g. ownership, cooperation between healthcare practitioners and trust) factors.

**Conclusion:**

This study shows that health system interventions have unpredictable paths of implementation. Discrepancies between planned and actual implementation reinforce findings in existing literature suggesting that while tools and defined operating procedures are necessary for any intervention, their successful application depends crucially on the context and environment in which implementation occurs. We anticipate that this evaluation will stimulate wider thinking about the implementation of similar models in low- and middle-income countries.

**Electronic supplementary material:**

The online version of this article (10.1186/s12913-017-2640-2) contains supplementary material, which is available to authorized users.

## Background

Access to medicines (ATM) has attracted increased global attention as a key component of universal health coverage. ATM has also been incorporated into national constitutions, as part of the Millennium Development Goals (Number 8e) [1] and more recently embedded in Sustainable Goal 3, which includes the target to reduce premature mortality from chronic diseases [2]. Furthermore, medicines have been identified as a key pillar of health systems [3]. Frameworks have been developed to represent ATM, with the most recent one by Bigdeli et al. proposing that ATM be viewed within a health system perspective [4]. However, challenges remain in low- and middle-income countries (LMICs) and reflect shortcomings in the health and supply systems in which medicines are distributed and delivered [1]. One key challenge is the pharmacy workforce shortage, which could undermine the performance of the medicines supply chain [5]; yet the growing burden of chronic disease demands efficient life-long medicines supply systems for patients.

In order to address some of these challenges, a novel centralized dispensing system has been applied to repetitive technical processes in the South African public sector, where dispensing services are contracted out to a private healthcare logistics company. In a previous article [6], we described the processes and actors involved in Chronic Dispensing Unit (CDU) implementation. In summary, the CDU collects prescriptions from over 200 healthcare facilities (hereinafter referred to as “facilities”) for about 300,000 patients with chronic illnesses each month, prepares individual patient parcels that are then distributed from either the facilities or from community distribution points [6, 7].

An article by Spinks et al. (2016) reported that to date, most automated dispensing innovations have been applied at a local level, such as hospital pharmacies or community pharmacies [8]. South Africa is among the few countries that have embarked on large-scale centralized dispensing in the public sector [8], however, little is documented on this intervention. In this article, we focus on the results from a process evaluation of the Chronic Dispensing Unit (CDU), the first large-scale automated dispensing system to be introduced in the South African public sector. The CDU was introduced in one province (Western Cape) in 2005 [6, 9, 10].

This qualitative evaluation was conducted in response to a request by the Western Cape Department of Health (WCDoH) in 2012 to inform process improvement. This study sought to compare what was planned with the actual implementation and to attempt to establish the causal elements and contextual factors influencing implementation.

## Methods

We used theory-driven evaluation (TDE) principles as outlined by Chen [11, 12] and Van Belle et al. [13] to guide this evaluation. Theory-driven evaluation as a methodological approach has the ability to contribute knowledge about how and why the intervention worked or failed; evidence that could be useful for understanding the intervention outcomes, for strengthening future implementation strategies, and for developing transferable lessons regarding barriers and facilitators to effective implementation [14]. A key principle of TDE is the development of a program theory, to explain how the planners or designers expect the intervention to be implemented and why it would lead to the desired outcome [15], which informs the choice and design of the intervention [13]. This detailing of assumptions is also referred to as the *action model*, which becomes a hypothesis that can be tested and further refined based on empirical findings [13]. Through this testing, the causal processes and the intervening contextual variables that produce change are referred to as the *change* model [13]. The *change model* provides the explanation of how and why the desired outcome would be obtained. To that end, the process of constructing the CDU’s program theory was inherent in the study design and was elicited through a process of document review, key informant interviews and a review of published literature [6]. In summary, the program theory was as follows: bringing together various actors and initiating appropriate implementation procedures within a supportive micro-, meso and macro-climate increases access to medicines. In this context, the actors were: the private sector with advanced logistical capability, a provincial implementation task team, frontline healthcare practitioners, community-based organizations, and a stable and adherent patient population. Increased access to medicine occurs by reducing pharmacists’ workload through relieving pharmacy staff from repetitive and time-consuming tasks; increasing time for patient-counselling; decongesting facilities and reducing patient waiting times. These factors ultimately contribute to improved health outcomes.

Since the focus of this study was to examine implementation dynamics, taking into account issues of context, a multiple, embedded case study design [16] was employed. We defined the case as the implementation of the CDU program and selected four facilities as the unit of analysis. Our study focused on urban facilities where CDU roll-out was first initiated, and was therefore deemed to be better established. Four primary facilities of different sizes were selected based on the monthly Patient Medicine Parcels (PMP) received from the CDU: <4000 (small); 4000–10,000 (medium) and more than 10,000 (large). For variability of experiences, we selected four sites: one small, two medium and one large.

### Data collection processes and tools

Theory-driven evaluation is flexible and methods-neutral: the choice of methods is informed by the study objectives [13]. Our data collection methods included: (a) in-depth interviews with representatives of selected actor groups [11], (Table 1); (b) a document review and (c) three feedback sessions with participants. The data collection was conducted during the period 2014–2015. All data collection tasks were conducted by the first author, a qualitative researcher with a background in public health.

**Table 1:** Study participants

Stakeholder group	Description and relevance to this study	Number of participants
*Implementing organization* (*Implementation task team for our purposes*): Provincial directors/managers, facility liaison officers and managers from the current and previous contracted service provider)	Responsible for organizing resources and coordinating implementation activities. The capability of this organization affects the quality of implementation.	8
*Implementers (actors):* mid-level managers, i.e. sub-structure pharmacists, primary healthcare managers; frontline healthcare practitioners - clinicians and health promoters	Responsible for implementation at the frontline.	32

#### Key informant interviews

Key informant interviews were conducted face-to-face at the respondent’s preferred location, with the exception of two whom we interviewed telephonically. Developing the program theory [6] guided the researchers’ understanding of how the intervention was supposed to work and informed subsequent interviews with key informants. We focused on the intervention as it had been implemented, i.e. the processes (e.g. patient selection and orientation, quality of prescriptions, feedback between the CDU and facilities, and management of non-collected medicines), contextual factors and intervention outcomes and recommendations for improving implementation.

Where possible, interviews were recorded; alternatively, notes were taken*.* Three participants refused to be recorded as a matter of preference. Once no new information was generated from the interviews (saturation), no further interviews were conducted.

#### Document analysis

We carried out a document review of program and policy documents and reports, including standard operating procedures and provincial chronic disease audit reports, in order to triangulate some of the respondents’ perspectives.

#### The feedback sessions with participants

Preliminary results were discussed with respondents in order to allow for member checking, an important validation technique in qualitative research [17]. The process was participatory: we presented at pre-scheduled provincial stakeholder meetings (in two cases) and organized a separate meeting (one case). This process allowed participants to engage with and contribute to our interpretation of results. Since this study was meant to contribute towards service improvement, knowledge co-construction with the intended users of the research was an important aspect of the research.

### Ethics

Ethics approval was granted by the Senate Research Committee at the University of the Western Cape and provincial government approval to conduct research in facilities was granted by the WCDoH. All participants were taken through the informed-consent procedure prior to interviewing and were also informed of their right to withdraw at any time without any consequences in accordance with the requirements of the Helsinki Declaration of 2008.

### Data analysis

The recorded interviews were transcribed verbatim. A hybrid approach of inductive and deductive coding and theme development was applied [18] to the analysis. This approach was appropriate because it is data-driven and uses pre-determined codes while also allowing for the addition of newer codes. Broad pre-determined codes were drawn from the components of the initial program theory covering CDU processes such as patient selection and management of non-collections. At the same time, emergent codes were identified during the analysis. The first author coded the data using Atlas. TI version 7 software. Reports were made for each facility, and a comparison looking at responses to similar questions by respondents from different facilities was done to ascertain how the initial program theory could be refined.

## Results

A key statement made by a senior member of the health directorate is fundamental to understanding both the successes and challenges of the CDU, particularly the influence of the health system within which the CDU is embedded:

*“The CDU is dependent upon a lot of interventions that collectively make the system, but if the building blocks [referring to the World Health Organization’s health system building blocks] are not in place, then it doesn’t matter how good the CDU package looks. [The]CDU is not a plaster that you stick on a wound. You’ve got to fix the building blocks, your [medical] depot has got to work, your staff and your facilities have to be present and working, your referral system has to work, the contract management for your medicines supply has to be done properly. It’s a complex system, but if the little bits are done, then cumulatively, the CDU works.”*

### Implementation successes and challenges

Key informants held overwhelmingly positive perceptions about the CDU and reported that the establishment of the CDU was a useful strategy to address prevailing barriers to accessing medicines. The majority indicated that the CDU was a part of their operational routine upon which they were dependent and without which the health system would be weakened.

Automation of the dispensing process was perceived by some informants as useful to improve the dispensing rate and relieve pharmacists of mundane dispensing tasks, as reported by two pharmacists:

*“If those 300,000 prescriptions [dispensed by the CDU monthly] needed to be done [manually], I can tell you, on a daily basis, the pharmacist can do only a 100 prescriptions. So, you can do the calculations. Even 200 prescriptions are a huge workload, even if we were able to pay salaries … and now we have an influx problem [with patients].”* (Sub-structure pharmacist manager).

*“When I worked in the facility in 2001 there was no CDU and you know we did six hundred scripts a day on our own, and when I returned to the system in 2008 there was this amazing system and it was just fantastic because it took that repetitive work away from the pharmacist - not doing the same scripts every month, you actually had a little bit more time to spend with the patients and actually answer their questions. It also reduces the pressure on the pharmacist. You know in the facilities there’s so much pressure on you that you eventually take your frustration out on the patient. So I think it has helped the pharmacists to reduce their workload and I hope that it makes us better pharmacists at the end of the day, able to focus more on the patient more than just focusing on getting that big pile of folders down.”* (Provincial pharmacist manager)

Although one of the quotes above suggests that the CDU created more time for patient counselling, there were also conflicting views. Either way, it seemed that the intervention increased the health system’s capacity to accommodate newly-diagnosed patients and allowed more time for the non-technical phases of dispensing such as face-to-face counselling that would have been difficult for pharmacists to fulfil without assistance.

All four facilities included in this study were reported to still have high patient volumes, with patient waiting times for medicine collection still reaching up to five hours at the largest facility. However, facilities with multiple distribution points or a separate “fast-track” queue for CDU patients managed to significantly reduce waiting times. This meant that CDU beneficiaries had separate queues from other patients at designated times in the facility or PMP were distributed from a separate building on the facility premises or in the community. One pharmacist stated that their facility was able to distribute in excess of 200 parcels within two hours. Whether the designated times for distribution were convenient for all patients remains unanswered.

Other benefits appreciated by healthcare practitioners included the flexibility of the dispensing system to accommodate special requests from facilities to dispense medicines for multiple months for mobile populations. This benefit was achieved in small increments from an initially rigid system to one with improved functionality.

In addition, one informant referred to the CDU as a *“control tower”,* capable of providing some information necessary for health planning and management such as prescribing practices, medicine usage trends, medicines expenditure per facility and identifying ‘clinic hopping’ patients with a tendency to collect medicines from more than one facility. Information on medicines expenditure for example was already used consistently for planning and monitoring.

Finally, the CDU program also encountered multiple challenges pertaining to contracting of suppliers and the operating context. The former, although outside the direct control of the intervention influenced its implementation significantly as highlighted below.

### Role of macro-level processes

#### Contracting of CDU service provider

The CDU service is a contracted service, whereby the appointed service provider is given a five-year term. At the time of the study, the service had been through one cycle of contractual change in 2011/12 and the second cycle was due in 2016/17. During interviews, many respondents mentioned how the first tender change-over disrupted the service greatly, while also presenting lessons for the future. One informant specifically described the first tender change-over experience as the *“…the straw that broke the camel’s back…”*, implying that the near collapse of the CDU during the first change-over process  exposed some weaknesses that already existed in the health system. This transition period was shrouded in controversy: service disruptions were caused by delays in the appointment of a service provider, loss of electronic patients’ records and a prolonged lead time required by the new service provider to become operational. Further details are provided in Additional file 1.

#### Role of contracting procedures on medicines supply

Delays in public sector medicines procurement were identified as a contributor to stock-outs of medicines that inadvertently affected the CDU’s operations. Delays were greatest during the supplier change-over periods where stock-outs rates rose from between 10-20 to 60-100 items at a time. Orders for CDU medical supplies are coordinated by the provincial depot and most purchases for the public sector are done at the national level. Many respondents cited the importance of investigating the reasons for delays in procurement and identifying interventions to address the challenges.

Stock-outs at the CDU resulted in PMP being dispatched without some essential medicines. When a PMP was dispatched to the facility without all the prescribed items, the local pharmacist would either dispense a suitable generic medicine if readily available in the pharmacy or an alternative under the authorization of a prescriber. This supplementary dispensing for patients registered with the CDU was not well received by healthcare practitioners as it created additional workload for pharmacy personnel.

### Implementation at the frontline

#### Planned vs. actual activities and results

In many instances, there were discrepancies between planned and actual activities as indicated in Table 2. Selected quotes from participants are provided in Additional file 2 to explain these discrepancies.

**Table 2:** Planned vs. actual activities and results

Dimension	Planned activities and expected results	Actual activities and results
Patient selection	Selection of stable patients	Selection of patients who are not clinically stable because strict guideline application proved difficult within a context of: (a) multi-morbidities (b) high prevalence of patients with sub-optimal outcomes (c) changing outcomes and (d) patients’ needs perceived to be beyond clinical care. In addition, non-medical factors such as service pressures, enrolment targets by management, intention to save on the facility’s financial budget for medicines by putting more patients on the CDU’s budget.
Prescription quality	Clinicians issue prescriptions in accordance with legislation and policies	Overall rate of prescription rejection was an estimated 4–5% (of approximately 14,000 prescriptions each day). Errors were attributed to: (a) cumbersome administrative processes attached to the intervention (b) misunderstanding of processes between healthcare practitioners and the service provider.
	Pharmacists check all new prescriptions for compliance with legislation and policies	Pharmacists did not always check prescriptions before submitting them to the CDU because they felt it was time consuming.
Dispensing and dispatch of patient medicine parcels (PMP)	Prescription verification, dispensing and delivery to the facility three working days before the collection date	Except when a prescription had been rejected for reasons earlier stated, PMP were delivered on time.
Medicines distribution	Pharmacist checks all parcels and fulfils the prescription requirements using pharmacy stock in case of stock-outs. Distribution of PMP follows at the facility or in the community.	Pharmacists did not check all parcels – the process was deemed to be time consuming and consequently to reduce the benefits of the intervention. Pharmacists recommended the use of transparent instead of opaque packaging and inclusion of prescriptions in the PMP to facilitate easier checking. That said, when there were stock-outs, the facility was provided with a list of outstanding prescriptions needs and these were fulfilled unless the facility was also stocked-out.
Health system causes for non-collected medicines	Patients are given 5 working days should they miss their scheduled appointment. Thereafter, PMP are returned to the CDU within 10 working days from the date of collection or the medication is absorbed into the facility’s pharmacy.	Challenges resulted from: (a) clinicians who were resistant to changing their ways of working to adapt to CDU requirements (b) locum doctors who were not familiar with processes (c) patients who reported for acute care prior to their CDU appointment often led to establishment of new appointment systems. Clinicians recommended marking CDU patient files differently from other patient files for easier identification.
Management of non-collected medicines	If a patient misses 2 appointments consecutively, the prescription is stopped and the patient must consult the clinician for counselling and assessment. Reports on non-collected PMP should be submitted to the CDU.	Some pharmacy staff returned non-collected PMP while others opened PMP that were not collected. The reasons given for the latter were: (a) shortage of space to keep the parcels until the patient comes or until the parcel is returned to the CDU (b) to discourage patients from missing appointments [coercion] Pharmacy staff who opened PMP believed that the same patients would come to the facility even if late so they could re-dispense medicines and save on their facility's financial budget for medicines. Unstable patients who missed appointments were not removed from the system as per protocol for similar reasons earlier mentioned (saving on facility budget and high prevalence of unstable patients).
Monitoring and Evaluation	Data on all activities	Mid-level managers found it difficult to comprehend routine data and in some cases doubted its accuracy. Statistics on collection of PMP were still under reported because healthcare practitioners considered reporting a time-consuming task and feared negative views.

#### Health system ‘hardware’ and ‘software’ influencing implementation

We expand on some aspects of the health system’s *‘hardware’* (including policies, training, appropriate infrastructure) and *‘software’* (including traditions, values, interpersonal interactions) because these contextual factors are key to understanding the implementation results presented above. With regard to infrastructure, limited storage space was a major determinant of how pharmacy personnel managed PMP at the facility. Referring to early days of implementation, one respondent said:

*“If you have a container of a 100 pills in your shelf it takes up a fairly small space on your shelf but when you take a 100 pills and divide that up into 20 you need space for 5 containers. The fridge items were also a huge problem because facilities had these little fridges for their own purposes. If you deliver a prescription which contains fridge items, you have to put the whole PMP into the fridge so the fridge becomes full. Facilities had to buy new fridges but didn’t have the budget.”* (Former Implementation Task Team member)

Limited storage space was further exacerbated by missed appointments by patients. One informant estimated that at least 40% of patients did not collect PMP during the initial phases of CDU implementation which created a challenge for facilities to keep non-collected parcels and at the same time create space for new stock. Over the years, the WCDoH facilitated installation of additional shelving in facilities. Also, improved shelving and storage, and clear labelling of parcels by the service provider led to improved retrievability of PMP. Previously, facilities had no system in place to organize the PMP, therefore, boxes containing PMP piled up and it took a long time for pharmacy personnel to locate PMP for distribution. Consequently, they opted to re-dispense from facility stock, which frustrated both healthcare practitioners and patients as patient queues grew longer.

Although we focused on urban facilities for this study, healthcare practitioners made some useful comparisons between urban and rural facilities with regards to preparedness for CDU implementation. They indicated that the patient load in urban facilities is much higher, hence the infrastructure demands are also high. Secondly, before CDU roll-out in some rural facilities, those facilities mimicked the CDU process on a local level by pre-packing a two-month medication supply for patients. As a result, minimal infrastructure and process adjustments were required when the CDU was introduced.

On the ‘software’ side, human interactions were key. Pressure from provincial management emerged as a factor that worked against facility preparedness for the intervention. Respondents collectively highlighted that the fast-paced roll-out to facilities was intended to meet patient enrolment targets. Also, the number of enrolled CDU patients at each facility was used as an indicator of good performance, which in part contributed to selection of patients whose suitability might be questionable.

Another issue, particularly in the early years of implementation, was inadequate orientation of health practitioners to the intervention. Because the CDU demanded the adoption of new administrative processes and new ways of working, an orientation to these processes and willingness of all actors to adopt new ways of working was necessary. An informant who was closely involved in the early years of implementation indicated that in the beginning, much attention was given to ensuring that the dispensing processes, the product and implementation protocols were in place but facility preparation was neglected:

*“The CDU could deliver a perfect product, but if it’s received in chaos, that product will also be seen as chaotic... I always say: in the first six months, when the CDU was implemented (2005-6), it did so much more harm to the reputation of the CDU than it did good.”* (Former member of the Implementation Task Team)

Facility preparation improved over time and standard operating procedures were revised owing to the lessons learnt from implementation in urban facilities. At the time of this research, the Implementation Task Team was conducting two to three training sessions at each facility prior to enrolment and regular follow-up from facility liaison officers was provided. Also, implementation has shifted to a phased approach to allow for more investment towards supporting facilities prior to and during implementation. Healthcare practitioners identified the implementation support offered by facility liaison officers as a strength of the intervention. The quote below illustrates positive relationships between healthcare practitioners and facility liaison officers.

*“Okay, what is working and needs to continue, it’s direct support from CDU like [name] and [name] they are playing a very excellent role actually, they are very important people and whoever is taking care of CDU parcels in the facilities has someone to phone, to talk to and then they've got those weekly schedules to visit facilities and check if everything is okay, providing training and support, this is very important to continue…”* (Senior manager, pharmacy services)

Finally, we identified the following set of essential elements for successful CDU implementation: ownership, trust, cooperation, communication, willingness to change and leadership. In Table 3, we provide some key informant voices and our own interpretations to illustrate the role of each of these elements.

**Table 3:** Essential health system ‘software’ elements

*Joint ownership: “When I went to various facilities to see what the problems were, the question that I kept asking myself was “who owns the CDU?”. In the facilities where the health workers were more collaborative … when that worked well, the CDU was implemented with less resistance. The problems were still there but they were resolved amicably. When it was only [regarded as] a pharmacy issue in the grand scheme of things … had nothing to do with the facility manager, the structure and the line function it didn’t work.”* (Implementation Task Team member)
*Trust: “… I always say have the name of the person first and always be consistent with that person and build a relationship with them because I know for me I just call [name of facility liaison officer]. [Name] knows what to do and by now you know how long it takes for [name] to get back to you because you have that trust.”* (Pharmacist, facility 4)
*Cooperation: “You find that in facilities, there is a disjuncture, with people working in silos, when you look at the CDU process, for example and how it’s supposed to work, it also requires team work in terms of the doctor, the nurse, the person in the pharmacy, the patient and often, you’ll find for example, you’ll end up having your chronic patient coming in for acute [care]getting another prescription when they are supposed to be coming in for another parcel but that’s because the people at work are not speaking to each other. I wonder how we can get these multi-disciplinary teams to work together for the system to work better than it is working at the moment because I think that some of the problems can be resolved in that way. Some of these non-collected parcels are not indicative of patients defaulting, it’s system issues.”* (Senior manager, WCDoH)
*Communication: “Yah you want to minimize the number of people involved (referring to involvement of locum doctors in the CDU process), because from the clinician’s perspective there is a lot of frustration because of that poor communication between different actors. I don’t know ‘Did the patient pick up their medication at the end of the month?’ the only way I know is if they have another appointment. So now what we have instructed them (locum doctors) is just to cross out the date for the next CDU appointment if we change the medication, so that’s one way to communicate to the pharmacist. That communicates to the pharmacy staff, don’t issue the parcel, the prescription has changed. Now, I don’t know if all pharmacy staff are aware of that.”* (Physician and Advisor to WCDoH)
*Willingness to change:* Changing some traditional practices e.g. in prescription writing was influenced by perceived individual and organizational benefits. When tasks were considered to be time consuming, there was a lack of motivation to do them.
*Leadership*: *“I have come to the conclusion that it’s the “captain of the ship” or the manager of the pharmacy who influences success. If he’s not performing well, then that pharmacy won’t function well”.* (former Implementation Task Team member)

## Discussion

Our findings suggest that the CDU has, to some degree, contributed to addressing barriers to access to medicines in the Western Cape Province despite many challenges faced during implementation. This study also adds to the limited body of work examining centralized dispensing models. Its strength lies in its ability to not only show how things worked well (or did not work), but also to identify elements that promoted the intervention’s success, and context factors that influenced implementers’ decisions at the frontline. Furthermore, it shows that health system interventions have unpredictable paths of implementation [19] as evidenced by a range of issues highlighted at the frontline and tied to how actors responded to both the intervention and contextual influences. In addition, there were macro-level influences, which were not anticipated at the design phase of the intervention.

### Understanding actor responses during implementation

While there were implementation guidelines and protocols, healthcare practitioners’ decisions were guided mostly by contextual realities. A typical example was how healthcare practitioners exercised discretion in patient selection rather than using standard criteria. Evidently, there were tensions between the ‘planned and the actual’ when assessing the action model. These tensions were caused by multiple factors including targets set by management, service pressures and facilities’ budgetary constraints.

In literature, healthcare practitioners have been identified as street-level bureaucrats [20], faced with the immediate consequences of new interventions and having to reconcile management’s demands for example, with the reality in the service delivery environment. In that sense, they have the ability to exercise discretionary power in either accommodating or resisting policy initiatives and in shaping them in ways that fit with their everyday realities [21]. Depending on the context and circumstances, healthcare practitioners’ exercise of discretionary power is not necessarily viewed negatively [22], as it might be what is deemed “best” in a particular situation rather than what is “right” in some absolute sense [23]. We reported in another study how contextual factors influenced healthcare practitioners’ decision-making despite the availability of guidelines [24] and we see a similar trend here.

Understanding actor responses also requires acknowledging the key elements that form the change model and influence implementation. In this study, *communication and cooperation between actors, willingness to change*, *ownership, leadership commitment and trust* were influential in driving the intervention’s success. This is evidence that relationships between actors are not purely ‘technical’, but are influenced by human dynamics [25]. Finally, there were also unexpected negative outcomes as a result of, inter alia, an oversight to acknowledge complexity [26] and macro-level processes such as those for procurement.

Overall, this study presents lessons for informing similar interventions. Key among these lessons was the use of a theoretical model that allows for a deeper level of explanation and offers a source of external validity [27], an approach that has been cited as important for understanding public health initiatives [28]. Since this was a process evaluation, findings from this study were discussed with actors involved in implementation, including the management. Some of the challenges identified such as clinicians’ difficulty to identify CDU patient folders; pharmacists’ request for transparent packaging (instead of opaque bags) for PMP and inclusion of prescriptions in PMP to facilitate easier quality assurance checks at facility level were well understood by the WCDoH and solutions were being explored. Of note however, although the case for use of transparent packaging for PMP was clear, management raised concerns about a potential breach of patient integrity and confidentiality rights hence alternative solutions were being sought. Also, macro-level challenges were acknowledged but require targeted interventions at national and provincial level.

### Implications for future research

Evidence on centralized dispensing is growing and urgently required to guide implementation in other settings. Some studies from Scandinavia have presented early implementation experiences [25]; and there are on-going debates in the United Kingdom about the introduction of centralized dispensing on a larger scale [8]. Although there is still much to learn, there is already some evidence that such interventions could improve dispensing efficiency and reduce dispensing incidents [29]. With increasing dispensing needs resulting from a growing burden of disease, there will be space for similar interventions and evaluations in LMICs. Our evaluation of CDU implementation in the Western Cape province of South Africa has relevance for thinking about process improvement and consideration of both ‘hardware’ and ‘software’ elements of the health system when planning similar interventions.

### Study limitations

We were unable to quantify the intervention’s intended outcomes (e.g. reduced pharmacists’ work load, patient waiting times) because these were not measured prior to and during implementation.

## Conclusion

This study shows that health system interventions have unpredictable paths of implementation. Clear differences between what was planned and actual implementation emerged in all facilities that were researched. The differences were primarily contextual, and a combination of ‘hardware’ (e.g. training and infrastructure) and ‘software’ (e.g. ownership, cooperation between healthcare practitioners and trust) issues. Our conclusion reinforces what some studies on implementation of health interventions have found, that while tools and standard operating procedures are necessary and valuable, their successful application depends crucially on the context and environment in which implementation occurs [30]. To our knowledge, this is the first article to evaluate the South African CDU and we anticipate that this theoretically-framed evaluation will stimulate wider thinking about the implementation of centralized dispensing models in other settings in LMICs.

## Additional files

Additional file 1: Textbox 1: Key events linked to the CDU’s first tender change-over process (2011/12). (DOCX 14 kb)

Additional file 2: Table S1. Selected quotes and observations to support views expressed in Table 2. (DOCX 16 kb)

## Abbreviations

CDU: Chronic Dispensing Unit
LMICs: Low-and-middle income countries
PHC: Primary Healthcare
PMP: Patient Medicine Parcel
TDE: Theory-driven evaluation
WCDoH: Western Cape Department of Health
