# Supplementary material for: Analyzing implementation dynamics using theory-driven evaluation principles: lessons learnt from a South African centralized chronic dispensing model
Source: BMC Health Serv Res. 2017 Dec 4;17(Suppl 2):724. doi: 10.1186/s12913-017-2640-2 (PMC5773901; doi:10.1186/s12913-017-2640-2)
Supplement: Supplementary file 1 — Textbox 1: Key events linked to the CDU’s first tender change-over process (2011/12). (DOCX 14 kb) [file 12913_2017_2640_MOESM1_ESM.docx]

**Additional file 1**

**Textbox 1: Key events linked to the CDU’s first tender change-over process (2011/12)**

| - **Delay in appointment of service provider**: To alleviate challenges, interim service providers including the first service provider, were appointed until a new service provider was appointed. - **Legal issue on intellectual property rights between the out-going service provider and WCDoH**: The provincial high court’s ruling was in favor of the service provider in accordance with the court’s interpretation of the service level agreement. However, there were consequences:   - data that had been electronically captured in the first five years of implementation were forfeited.   - the outgoing service provider handed over hard copies of prescriptions (for over 200 000 patients) to the WCDoH. The complex data transfer process had further implications on the next service provider’s ability to efficiently continue the dispensing service in the initial phase. - **The next service provider took longer than anticipated to adjust to service requirements**: This was a result of the issues stated above and was further compounded by the implementation of new business processes and commissioning of new equipment. In addition, the demand for the service was much higher than had been anticipated, which resulted in omitted or late deliveries.   The impact was felt at the facility level, increased patient waiting times were experienced, undoing some gains that had been achieved. It took some months for the service to stabilize again.   - **WCDOH’s response**:   The Department issued press statements to alert patients of the situation, set-up a temporary telephone helpline for patients to call with queries and instituted interim measures to ensure that patients obtained medicines until the system had stabilized again. For the first weeks of the transition, the new service provider and WCDoH jointly reverted to manual dispensing. WCDoH also suspended some facilities from the CDU for about three months to allow the service to stabilize again. |
| --- |
