# Supplementary material for: Analyzing implementation dynamics using theory-driven evaluation principles: lessons learnt from a South African centralized chronic dispensing model
Source: BMC Health Serv Res. 2017 Dec 4;17(Suppl 2):724. doi: 10.1186/s12913-017-2640-2 (PMC5773901; doi:10.1186/s12913-017-2640-2)
Supplement: Supplementary file 2 — Selected quotes and observations to support views expressed in Table 2. (DOCX 16 kb) [file 12913_2017_2640_MOESM2_ESM.docx]

# Additional file 2

## Table S1: Selected quotes and observations to support views expressed in Table 2.

| Patient selection  *Selection based on budget:* *“The other thing that makes us (to) put patients on CDU is to do with the budget. We have a different budget for patients on CDU which comes from provincial level and we have our own budget serving the patients coming through the facility. So the more people we get on CDU the more we can save on (our) budget. The facility budget is not increasing and the patients are increasing every year and we encourage the clinicians to put patients on CDU”*. (Pharmacist, facility A)  *Patients’ needs perceived to be beyond clinical care: “Health can do so much, inter-sectoral collaboration is required because poverty plays a role, your circumstances, economic factors and all of that.”* (Senior Manager, WCDoH)  *Service pressures: “…definitely there are people who are put on CDU who are not completely within the criteria of controlled blood pressure, controlled blood sugar. You got people who are fairly controlled but not perfectly controlled and if you have to see everybody every month who have their reading abnormal, it is not practical.”* (Physician 1, facility C)  *Service pressures: “It happens also that we would like to decongest facilities, the doctors sometimes are putting patients on CDU sooner than the patient is really stable, just for the sake of obtaining medication from CDU…the patient is not yet very compliant maybe he needs more checks on the blood pressure, or glucose levels.”* (Senior Manager, Pharmacy services)  *Changing outcomes:* *“I think that the assumption is that if the doctor puts them (patients) on a six-month prescription then the blood pressure is going to be stable for the entire six months, which you can’t be sure of.”* (Physician 2, facility C)  *Varying perspectives on what is appropriate:* *“It depends on the clinicians, some clinicians don't have a problem of putting someone (on CDU) who has a diastolic of 110, I’ve seen it and it makes no sense to me.” (Physician and Advisor to WCDoH)* |
| --- |
| Prescription quality  *Poor prescription writing:* *“…we’ve got prescribers who aren’t always writing legible prescriptions and then pharmacists are just assuming that they can read it and therefore this person at the CDU is going to be able to read it and I’ve captured some scripts and I was alarmed at how poor our prescriptions looked.” (*Implementation Task Team member)  *Deliberate decisions by clinicians:* *Yah that whole communication between clinician and pharmacy I must say in other facilities you get away with “murder” …you know they (clinicians) don't stick strictly to the code list restrictions... I don't know if I should admit this (laughing) but the doctors and specialists are getting away with prescribing (items that are not on the code list) …”* (*Physician and Advisor to WCDoH*)  *Prescription writing considered a cumbersome task:* “*That is why I have arthritis in my right shoulder because of writing a lot and especially now we don't have stickers (with pre-written titles). You have to write the name the surname, the folder number, the age, the ID number. You have to write everything down and it is very easy to make a mistake. After seeing 30-40 patients you can make mistakes very easily. That is a bit of a problem I must say and I don't know when is this going to end, not having stickers is a big problem.* (Nurse, facility D)  *Pharmacists not checking prescriptions*: *“I usually don't get the time to check all the scripts. I’m the only Pharmacist and the 2 assistants. I will not check each and every script especially with patients waiting outside.”* (pharmacist, facility D)  *Rate of prescription errors*: *“…we do about 14 000 scripts a day, and between four to five percent of that will be rejected every day. So that’s quite a substantial amount…150 scripts minimum a day are rejected, so that’s 150 patients that on the next collection day will not have a parcel waiting for them.”* (Implementation Task Team member) |
| Quality assurance  *“…you still find scripts that are not dispensed by CDU, you still find those that the patient didn’t receive*  *a parcel, so they ask us to fill the query form, but there is no time, that’s extra admin work... why don’t they*  *have a quality assurance system to check on the pharmacy?”* (Pharmacist, facility D) |
| Medicines distribution  Missing PMP: *“…they (facilities) report that they didn’t receive a PMP (parcel) then when we investigate we see that it was delivered to this site and someone signed for it, and then they phone back, and say ok we found it. So it could also be how they are managing the process at the facility and you can see there is a common pattern in some of the facilities. Some of the facilities will keep them in the box with all the details and all the paper work in there so it makes it easy to maintain. Some of the facilities will take it out of the boxes that we have provided and pack it, and then it becomes a little bit more difficult to find. (Name of Implementation Task Team member) is really working hard at teaching best practice, some facilities have got a fantastic way of organizing parcels, they put up manifests, patients come in and they say: “I am here to fetch my parcel it’s (in) box 653.”*” (Implementation Task Team member)  Case 1 (facility C): On the 26^th^ of February 2014, the pharmacist assistant (also CDU champion) reported 16 missing PMP (i.e. not delivered by the CDU) for that day’s distribution. The researcher followed up on this case and two days later, the pharmacist assistant reported that only 5 were actually missing (for reasons unknown at that stage). The other 11 had been reported as missing as a result of facility errors i.e. patients had been given wrong dates (for 10) and for the 1, the prescription had not been sent to the CDU. |
| Non-collected medicines policy: Why some pharmacists either return parcels earlier or absorb stock into facilities.  Why absorb rather than return PMP? *“Some patients do come to collect their medicines later (after their appointment date) so we need the medicines otherwise we will go over our budget.”* (Pharmacist, facility C)  Why return PMPs earlier than 10 working days (every second day)*:* *“We try to return the manifest every second day because of resource constraints… In any case the number of patients who will come is 2-3 so it makes little difference.”* (Pharmacist, facility C)  Observations from the dispensary: *“We see it here we get stuff (PMP) back, not five days after, we get it the next day. Yah so they just, at the end of the day say these are the patients that didn’t collect, they put it all into one box, tape it up and the next day our driver comes they say there you go take it out because they want space. There is no space in these facilities.”* (Implementation Task Team member) |
| Monitoring and evaluation information barely usable from the managers’ perspective: *“…we need to revisit the information that we are getting, the information is unmanageable, reports are very difficult (to interpret). The system is not user friendly and with time constraints people will not go there, that is one thing… If we could draw results by your facility, that could be helpful.” (Sub-structure manager, Pharmacy Services)*  Need for better patient monitoring: *“It also comes back to chronic disease management. We should start off by having chronic disease registers so that we have a code that these patients are on CDU and we know what is happening (to them).* *(Sub-structure manager, Pharmacy services)* |
